# RoCoLe: A *robusta* coffee leaf images dataset for evaluation of machine learning based methods in plant diseases recognition

**DOI:** 10.1016/j.dib.2019.104414

**Published:** 2019-08-19

**Authors:** Jorge Parraga-Alava, Kevin Cusme, Angélica Loor, Esneider Santander

**Affiliations:** a Escuela Superior Politécnica Agropecuaria de Manabí Manuel Félix López, Calceta, Ecuador; b Universidad de Santiago de Chile, Santiago, Chile

**Keywords:** Machine learning, Plant diseases recognition, Coffee leaf rust, *Hemileia vastatrix*, Red spider mite, *Tetranychus urticae*

## Abstract

In this article we introduce a *robusta* coffee leaf images dataset called RoCoLe. The dataset contains 1560 leaf images with visible red mites and spots (denoting coffee leaf rust presence) for infection cases and images without such structures for healthy cases. In addition, the data set includes annotations regarding objects (leaves), state (healthy and unhealthy) and the severity of disease (leaf area with spots). Images were all obtained in real-world conditions in the same coffee plants field using a smartphone camera. RoCoLe data set facilitates the evaluation of the performance of machine learning algorithms used in image segmentation and classification problems related to plant diseases recognition. The current dataset is freely and publicly available at https://doi.org/10.17632/c5yvn32dzg.2.

**Table:** Specifications Table

Subject area	*Computer Science; Agricultural and Biological Sciences*
More specific subject area	*Agronomy and Crop Science; Computer Vision and Pattern Recognition; Disease recognition*
Type of data	*Images, Annotation files.*
How data was acquired	*Images were acquired using a 5MP, f/2.2 autofocus smartphone camera. Annotation data were manually generated using a data labeling web-tool.*
Data format	*Data format for images are jpeg; annotation data are collected in JSON, CSV, COCO, VOC and XLSX format.*
Experimental factors	The images were taken at a working distance of 200–300 mm from coffee plants during cloudy, sunny and windy days and considering scenarios with background variety. Images correspond to upper and back sides of healthy and infected leaves.
Experimental features	*Infected and healthy images of coffee tree leaves were acquired using a smartphone camera. To set the rust infection severity, we have adopted the OIRSA (Organismo Internacional Regional de Sanidad Agropecuaria) method*[1]*which was proposed by the Laboratorio de Análisis de Riesgo Epidemiológico Fitosanitario (LANREF), where four diseases level are individually incorporated as annotations over the image. The ground-truth of coffee plants diseases presence as well as their severity were assessed by visually by an expert.*
Data source location	*Ciudad de la Investigación, Innovación y Desarrollo Agropecuario (CIIDEA), ESPAM MFL, Calceta, Manabí, Ecuador. Latitude -0.834206 and Longitude -80.177159.*
Data accessibility	Data is publicly available on mendeley data public repository with 10.17632/c5yvn32dzg.2 doi, at https://doi.org/10.17632/c5yvn32dzg.2

**Table:** 

**Value of the data** • Data can be used for training, testing and validation of classification algorithms for binary and multiclass problems using images of healthy leaves or with red spider mite presence and images with rust infection severity, respectively. • Data can be used for benchmarking of algorithms for coffee plants diseases recognition and diagnosis as well as for images segmentation. • Data can server as a motivation to encourage further research into plant diseases and machine learning methods for coffee pest identification. • Images in dataset include annotations of the ground-truth for objects (leaves, red spider mite presence, rust presence and infection severity) which can be used to improve the accuracy of classification/segmentation image algorithms trained on this dataset as well as to extract new knowledge about diseases that affect the leaves of coffee plants.

## 1 Data

**Fig. 1:**
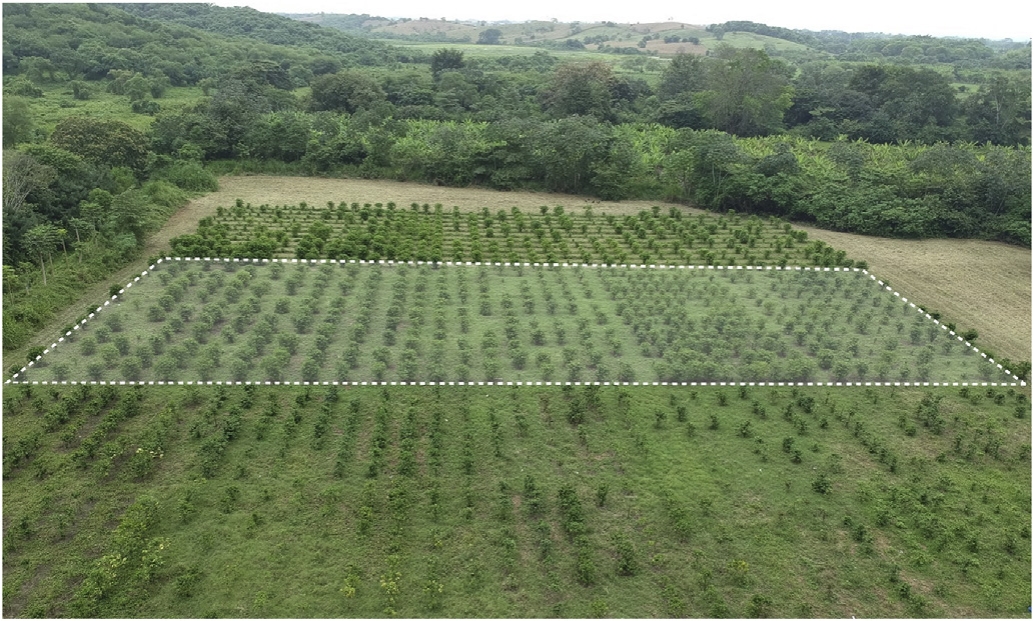
The aerial view of the coffee plants in the farmland of the study area (CIIDEA, Calceta, Manabí, Ecuador). In dashed line zone, we highlighted plants used to obtain images. The empty spaces correspond to not considered plants because they were plants growing process or unseeded space.

The *robusta* coffee leaf images dataset (RoCoLe) provides images that can be used to train and validate the performance of machine learning algorithms used in binary and multiclass classification problems as well as in segmentation tasks specially related to plant diseases recognition. The RoCoLe dataset contains imagery of upper and back sides of coffee leaves collected from *robusta* coffee crops showing different states (healthy and unhealthy) as well as presence of disease (rust^1^ and red spider mite^2^ presence) and infection severity. A total of 4 images are included per each one of the 390 coffee plants available in the study area (Fig. 1). Thus, in total, 1560 images of *robusta* coffee leaves are included in the data set.

## 2 Experimental design, materials, and methods

The coffee plants images of healthy and unhealthy leaves were acquired using a 5-MP smartphone camera at a working distance of 200–300 mm without zoom. The images were taken daily under real conditions such as multiple lighting brightness (cloudy, sunny and windy days), backgrounds (other plants and weed) and temperature levels (high and low humidity levels) to have real and representative samples of coffee plants. In the study area, we considered a crop with 390 coffee plants. On each coffee plant in the crop, we taken images of upper and back sides of healthy and infected leaves which yielded the total of 1560 images.

To provide to RoCoLE the capacities to evaluate the performance of the mentioned and other machine learning methods, we carried out an annotation data process to obtain a fully-labeled of the 1560 coffee leaf images using a data labeling web-tool called ©Labelbox. Two types of annotations were made: (1) object segmentation and (2) classification.

In machine learning area, object segmentation aims separating parts of an image into pieces which are conceptually meaning. Methods as artificial neural networks, genetic algorithms and clustering have been used to perform segmentation tasks [2], [3], [4]. Meanwhile, classification aims finding a class to which an item belongs as from patterns in a labeled data set. Here, methods as artificial neural networks, SVM (Support Vector Machine) and decision trees have been used to perform classification tasks [5], [6], [7].

Our dataset includes annotations of object segmentation, where the labeler recognizes and segments each object (leaf). Each object was further annotated as healthy or unhealthy. For annotations of classification, the leaf was labeled as healthy, red mite presence, rust level 1, rust level 2, rust level 3 or rust level 4. To set the rust infection severity we consider the OIRSA method [1]. It is summarized in Table 1.

**Table 1:** Severity scale of rust in coffee leaf.

Level	Affected leaf area (spots)
1	1–5%
2	6–20%
3	21–50%
4	>50%

Table 1 shows the descriptions of the levels or severity grades of the rust. On annotations, each level is set according to the percent of affected leaf area. For instance, when the number of spots is more than half the leaf, the image is classified with the maximum level, i.e., rust level 4. Infection severity up to such level is considered, because we used the OIRSA method.

The fully-labeled image dataset can be used for reproducible research where machine learning methods are used to tackle segmentation and classification problems.

**Fig. 2:**
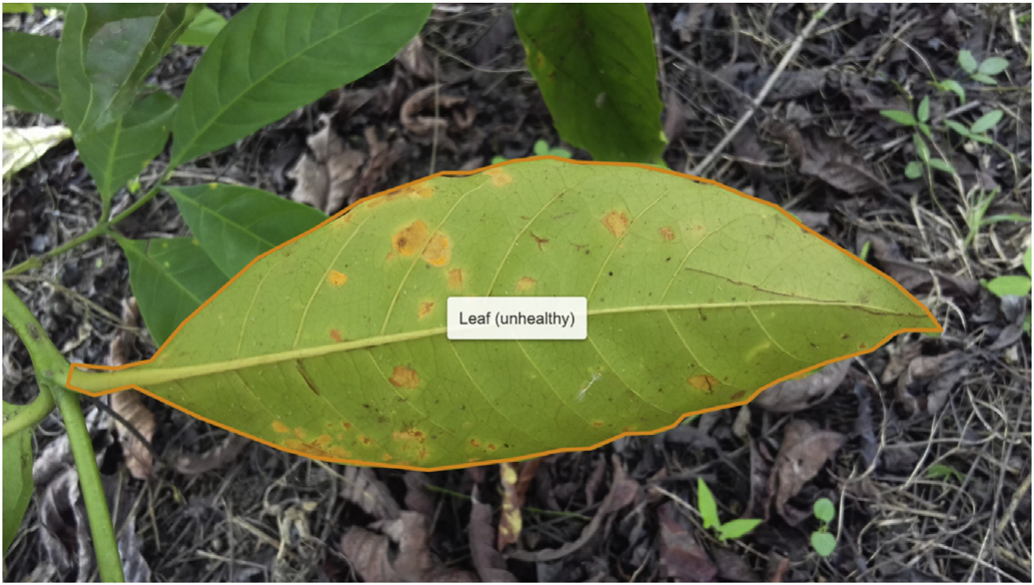
Annotation examples of a segmentation mask in RoCoLe dataset.

For segmentation problems, RoCoLe provides object annotations to segment the leaves (Fig. 2).

Fig. 2 shows an example of the visual appearance of annotated images for segmentation purpose. Here, an object is identified through a mask or an orange limited-area denoting the segment in the image where a leaf is located. Moreover, it also includes the type of object, i.e., unhealthy leaf.

**Fig. 3:**
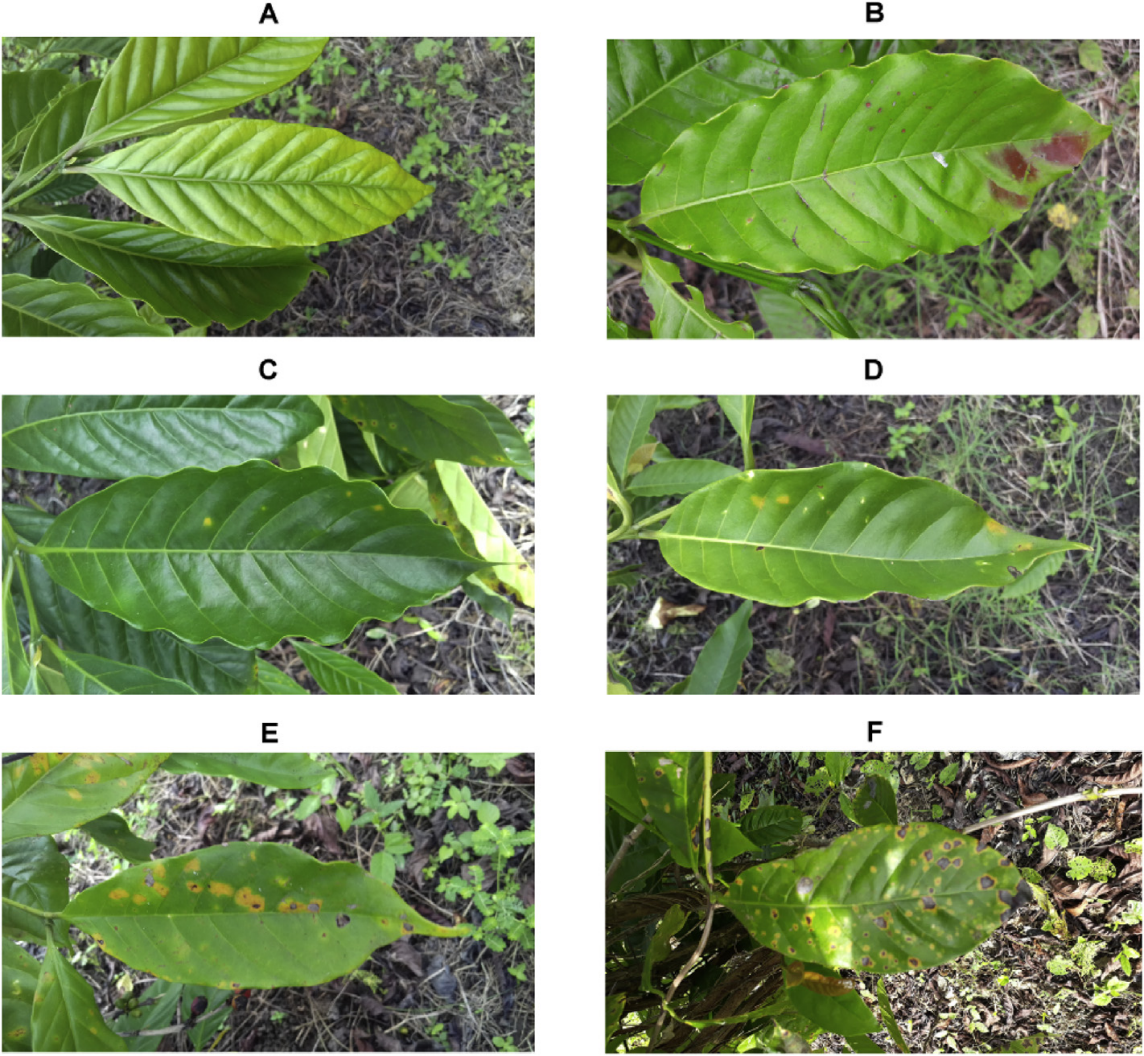
Example of coffee leaf with different states (classes). A) healthy. B) Red Spider Mite. C) Rust level 1. D) Rust level 2. E) Rust level 3. F) Rust level 4.

For classification problems, RoCoLe provides labels of six classes: healthy, red spider mite presence, rust level 1, rust level 2, rust level 3 and rust level 4 (Fig. 3). For each one, there are 791, 167, 344, 166, 62 and 30 images, respectively.

Fig. 3 shows examples of the upper side of leaf images for each class. From this type of image, the labels are set by performing a visual inspection to establish the leaf state. All this labeled process is validated by an expert.

## Footnotes

1 Rust is a disease caused by the hemileia vastatrix fungus.

2 The tetranychus urticae is a specie of plant-feeding mite. It is commonly known as red spider mite.
